# Restore intestinal steady-state: new advances in the clinical management of chemotherapy-associated diarrhea and constipation

**DOI:** 10.1007/s10735-025-10367-w

**Published:** 2025-03-08

**Authors:** Miaoqi Chen, Yamao Li, Peijun Chen

**Affiliations:** 1 Imperial College of London, London, UK; 2 Ningxia Medical University, Yinchuan, China; 3 Yancheng Sixth People’s Hospital, Yancheng, Jiangsu China

**Keywords:** Tumor, Chemotherapy, Intestinal flora, Chemotherapy-induced diarrhea, Chemotherapy induced constipation

## Abstract

Chemotherapy remains the primary therapeutic strategy for most tumors, particularly those at advanced stages with distant metastases and resistance to molecularly targeted therapy or immunotherapy. There are many manifestations of chemotherapy-induced gastrointestinal toxicity (CIGT), including chemotherapy-induced diarrhea (CID) and chemotherapy-induced constipation (CIC). Although the World Health Organisation and the International Association Against Cancer have different grading criteria and strategies for the prevention and treatment of CIGT, there are still many unanswered questions that need to be clarified. This review critically describes pathological mechanisms and clinical research, analyzing the variability in diagnostic criteria and the absence of standardization in grading severity. We identify a critical gap in understanding the molecular underpinnings of CID and CIC and suggest targeted areas for future research, including developing personalized treatment approaches based on genetic profiling. The findings suggest a comprehensive treatment approach combining pharmacological and non-pharmacological strategies to enhance life quality and treatment adherence. This review will offer a comprehensive bird-eye of pathophysiological mechanisms, clinical manifestations, and therapeutic strategies of CIGT, thereby enriching accessible references to clinicians, and helping them to prevent and control CID and CIC.

## Introduction

**Fig. 1:**
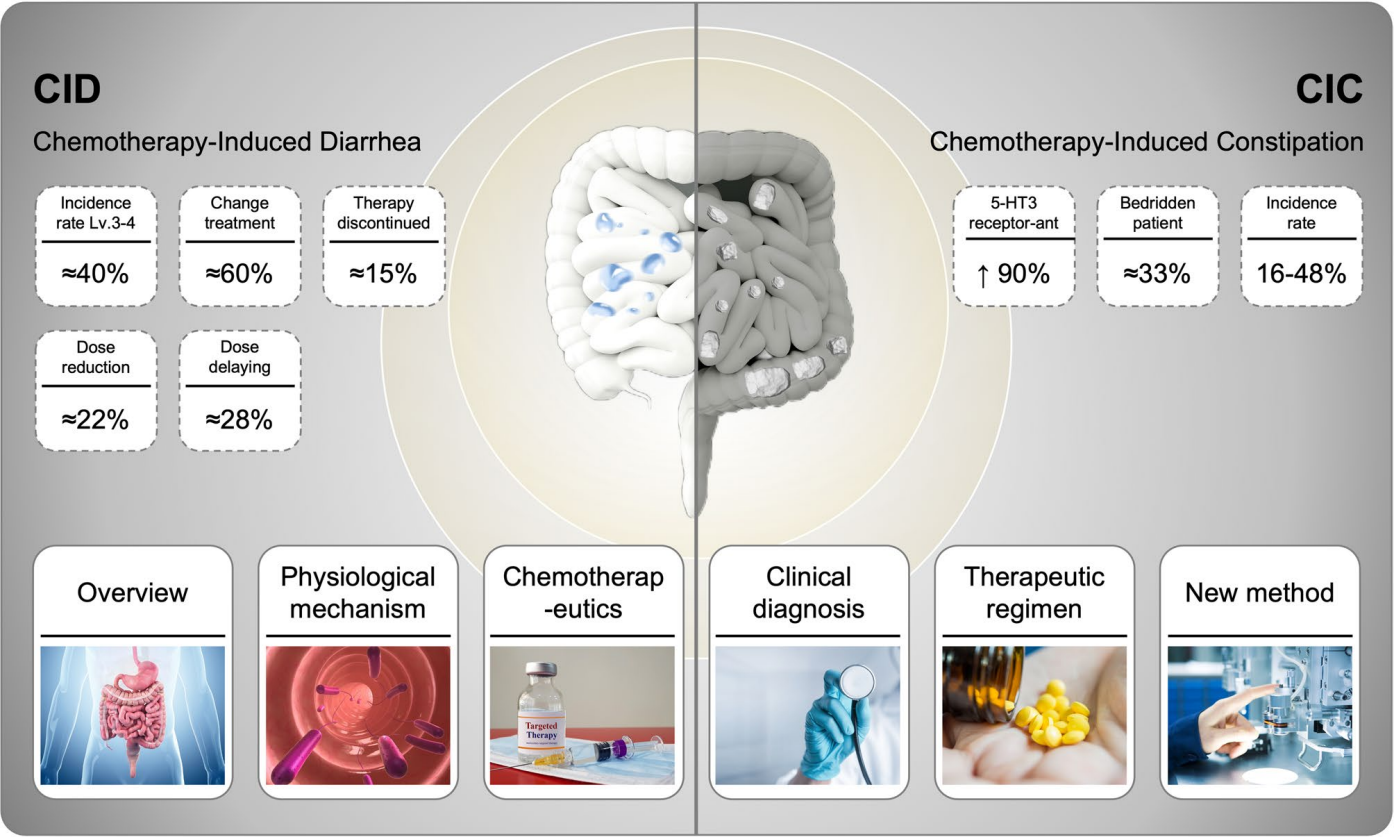
Schematic content outline for a review of chemotherapy-related diarrhea and constipation. The incidence of CID, treatment adjustment, and dose adjustment, were shown on the left. On the right are CIC data, including the proportion of 5-HT3 receptor antagonists used and the proportion of bedridden patients. The middle diagram shows how the gut behaves differently in CID and CIC states

Chemotherapy is currently the primary treatment for most tumor patients, especially patients at advanced stage, accompanied by distal metastases and resistant to molecularly targeted therapy or immunotherapy. Chemotherapy-induced gastrointestinal toxicity (CIGT) is a common complication of chemotherapy, the incidence and severity of which are associated with the type of chemotherapeutic agent, dose, mode of administration, and baseline characteristics of patients. CIGT manifests itself in a variety of ways, including diarrhea, constipation, nausea, vomiting, loss of appetite, and other clinical symptoms, and in severe instances, it can result in dehydration, electrolyte disorders, malnutrition, etc., which can significantly impact the quality of life, and even threaten the safety of patients’ lives (Zraik and Heß-Busch 2021; Goodman 1989). Figure 1 provides a review of chemotherapy-related diarrhea and constipation and illustrates the main clinical features of CID and CIC, including incidence andtherapeutic impact.

Chemotherapy-induced intestinal toxic side effects include chemotherapy-induced diarrhea (CID) and chemotherapy-induced constipation (CIC). Currently, the World Health Organisation and the International Society Against Cancer have different grading criteria for CID, which leads to an unclear diagnosis of CID, and there is no consensus on the pathological, physiological, and molecular mechanisms of CID. Additionally, there is no grading standard for CIC in oncology patients, and clinical treatment often uses enemas and suppositories, which are not suitable for patients with advanced stage tumors. Relevant reports indicate that the clinical incidence of grade 3–4 level of CID is about 40%, the clinical incidence of CIC is about 16–48%, and the use of antiemetic drugs can increase the incidence (Fig. 1).

Chemotherapeutic agents can kill tumor cells while producing toxic side effects on normal immune cells, leading to a significant decrease in immunity in patients with cancer. Chemotherapeutic drugs also damage intestinal epithelial cells and leading to dysbiosis, the above problems limit the development and application of inexpensive chemotherapeutic drugs in terms of both availability and the use of chemotherapeutic drugs regarding dosage and patient. Furthermore, the symptoms of diarrhea and constipation in oncology patients may be triggered by a variety of factors, which can lead to the overlooking of pharmacogenetic triggers. Pre-existing gastrointestinal conditions, such as inflammatory bowel disease (IBD), can also significantly alter the severity and presentation of CID and CIC.

To solve these problems, the study of CIGT should not only focus on the mechanism of its occurrence but also on how to alleviate its effects on patients and improve their life quality. Various methods and strategies have been developed, yet numerous challenges remain. These includes accurately assessing patient risks, choosing the most appropriate prevention and treatment regimen, and evaluating the effectiveness of treatment. This article intends to offer a comprehensive review of pathophysiological mechanisms, clinical manifestations, and therapeutic strategies of CIGT, offering clinicians with additional reference information to better manage CID or CIC.

### CIGT overview

About 40% of all patients taking chemotherapy drugs will develop a series of gut-related toxicity, and the probability of this toxicity increases with higher doses of chemotherapy drugs, up to 100%. (Dahlgren et al. 2021) The clinical side effects of chemotherapy include nausea, vomiting, abdominal pain, constipation, diarrhea, weight loss, and mucosal ulcers (Sonis 2004). Table 1 presents the classification of side effects, including diarrhea, constipation, nausea, and mucosal ulcers, based on severity.

**Table 1:** Common chemotherapy-associated gastrointestinal toxicity scoring standard, from Common Terminology Criteria for Adverse Events (CTCAE), version 5.0. Health NIo. Common Terminology Criteria for Adverse Events (CTCAE). 2017

Side effects	Grade 1	Grade 2	Grade 3	Grade 4	Grade 5
Diarrhea	Increase of < 4 stools per day over baseline	4–6 stools per day; affects daily activities	≥ 7 stools per day; hospitalization	Life-threatening; urgent intervention	Death
Constipation	Intermittent symptoms; occasional remedies	Persistent symptoms; regular remedies	Severe constipation (Obstipation); manual intervention	Life-threatening; urgent intervention	Death
Nausea	Loss of appetite	Reduced oral intake without significant side effects	Inadequate intake; nutritional intervention needed	–	–
Abdominal pain	Mild	Moderate	Severe	–	–
Vomiting	No intervention	Outpatient IV hydration	Nutritional support intervention	Life-threatening	Death
Weight loss	5 to < 10% from baseline; no intervention	10 to < 20%; nutritional support	≥ 20%; intensive nutritional support	–	–
Mucosal ulcers	Asymptomatic; no intervention	Symptomatic; altered GI function	Severely; intensive care	Life-threatening; urgent surgical intervention	Death

The occurrence of CID and CIC is closely related to the postoperative recovery of patients. As one of the common intestinal toxic side effects, CID affects an estimated 50–80% of cancer patients and up to 10 percent of patients suffer from severe type, especially those with advanced cancer, where CID can significantly deepen their pain (Stein et al. 2010). CID severely interferes with anticancer therapy, with about 60% of patients modifying their treatment regimen as a result, 22% moderately reducing their dose, 28% delaying their dose, and 15% discontinuing treatment altogether due to the distress of CID. Moreover, some patients who adhere to their treatment experience CID symptoms persisting for up to 10 years after treatment (McQuade et al. 2016).

CIC is often overlooked as one of the effects of CIGT (Belsey et al. 2010). Normally CIC has an incidence of 16–48%, the incidence of constipation varies from 50 to 78% in people with advanced tumors, 16% in patients receiving chemotherapy with cytotoxic drugs, with 5% experiencing severe constipation (Pehlivan and Nural 2022; Gaertner et al. 2015). The incidence of constipation rises to 90% when antiemetic drugs are used concurrently to relieve digestive symptoms (McQuade et al. 2016; Escalante et al. 2017_)._ It has been suggested that about one-third of cancer patients receiving chemotherapy experience CIC, negatively affecting their quality of life (Pehlivan and Nural 2022).

Severe complications, including toxic megacolon and intestinal perforation, have been reported in IBD patients receiving chemotherapy. Chemotherapy could lead to acute IBD exacerbations, requiring urgent clinical interventions and hospitalization (Pellegrino et al. 2023).

It is evident that a series of intestinal toxic reactions after chemotherapy have strong obstacles to patients’ normal lives, and it is urgent to find ways to alleviate such toxic effects. The diagnosis and improvement measures of CID and CIC are less common and equally important than those in studies of intestinal mucositis.

### Mechanisms leading to CID and CIC

#### Mechanisms leading to CID

CID typically occurs on the day of or after chemotherapy treatment and can last for about 7 days. Typical clinical manifestations are painless diarrhea or mild abdominal pain during chemotherapy, mostly watery stools, up to tens of times a day. To date, the mechanism of CID remains unclear (Okunaka et al. 2022), and studies have demonstrated previously that CID involves a variety of factors, including intestinal epithelial damage, inflammation, infection and antibiotic use. Histopathological evidence suggests that CID may involve multiple physiological or pathological processes, resulting in an disruption of absorption and secretion in the small intestine, these processes vary according to the causative factors (Kornblau et al. 2000). Additionally, disruption of intestinal tight junctions and dysfunction of mucosal barrier may also cause potential mechanisms of diarrhea (Wardill and Bowen 2013).

After chemotherapy, the apoptosis of intestinal crypt cells significantly increases, rising up to 7 folds, while the area of villus, length of the crypt, mitotic count of each crypt, and height of intestinal cell decrease within 3 days (Keefe et al. 2000). This apoptosis in crypts has effects on the intestinal mucositis intestinal mucositis, leading to hypoplastic villi atrophy and a decline in intestinal cell height following crypt cell apoptosis (Keefe et al. 2000). Chemotherapeutic agents bring direct cellular damage, causing superficial necrosis and disruption of homeostasis between villus and crypt cells on the intestinal epithelia. This damage leads to intestinal dysfunction, characterised by an imbalance of absorption and secretion (Abraham and Sellin 2007; Ratnaike and Jones 1998). In addition, the pathology of CID development is strongly associated with inflammation. After the administration of irinotecan, pro-inflammatory cytokines (TNF, IL-1β, IL-6) are expressed at multiple sites throughout the digestive tract. These pro-inflammatory cytokines are key drivers of mucositis, and their spikes in tissue staining coincide with the early signs of pathological changes and the onset of diarrhea (Logan et al. 2008).

The symbiotic microbial composition of the microbiome changes after chemotherapy exposure, which can affect barrier function and damage the gut, thereby the protective effect of symbiotic microbes on intestinal integrity (Stringer et al. 2009).

#### Mechanisms leading to CIC

CIC is a form of constipation characterized by a prolonged stool interval, hardening of stool, and shape change in patients receiving chemotherapy drugs and adjuvant therapies. Patients with severe CIC often have abdominal distension and paroxysmal abdominal pain. Constipation exacerbates the gastrointestinal symptoms, causing abdominal distension and aggravates gastrointestinal reactions such as nausea and vomiting. In addition, straining to defecate would increase the abdominal pressure over a prolonged period, which reduces the volume of returning blood and simultaneously increases the intracranial pressure continuously. This may lead to malignant cardiac arrhythmia and cerebrovascular accidents, prolonged squatting during defecation can also cause numbness of the legs and feet, and dizziness that leads to accidents of falling. The dry stool can cause anal fissures and hemorrhoids, while intestinal stool block accumulation can affect the absorption of chemotherapy drugs (Bharucha and Lacy 2020). Without timely treatment, more serious complications may arise, such as intestinal obstruction. The obstructed stool can increase local pressure in the intestinal lumen, leading to wall ischemia, mucosal necrosis, pain, bleeding, and perforation, and eventually lead to reduced, delayed or stalled chemotherapy for tumor patients, potentially life-threatening (Escalante et al. 2017).

Some studies indicated CIC as effect closely linked to the effect of drugs on intestinal nerve endings. The enteric nervous system co-innervates the gastrointestinal tract, influencing intestinal motility and defecation. Studies in an animal model of CIC in 5-fluorouracil-treated mice has demonstrated that the absence of intestinal muscularis propria neurons leads to delayed gastrointestinal transit and suppressed colonic contraction (McQuade et al. 2016), the absence of intestinal neurons following treatment with oxaliplatin, and cisplatin application was significantly associated with reduced colonic motility and extended gastrointestinal transit time. Chemotherapeutic drugs such as Vincristine analogues, which are neurotoxic, lead to a decrease in gastrointestinal smooth muscle tone and reduced gastrointestinal motility.

**Fig. 2:**
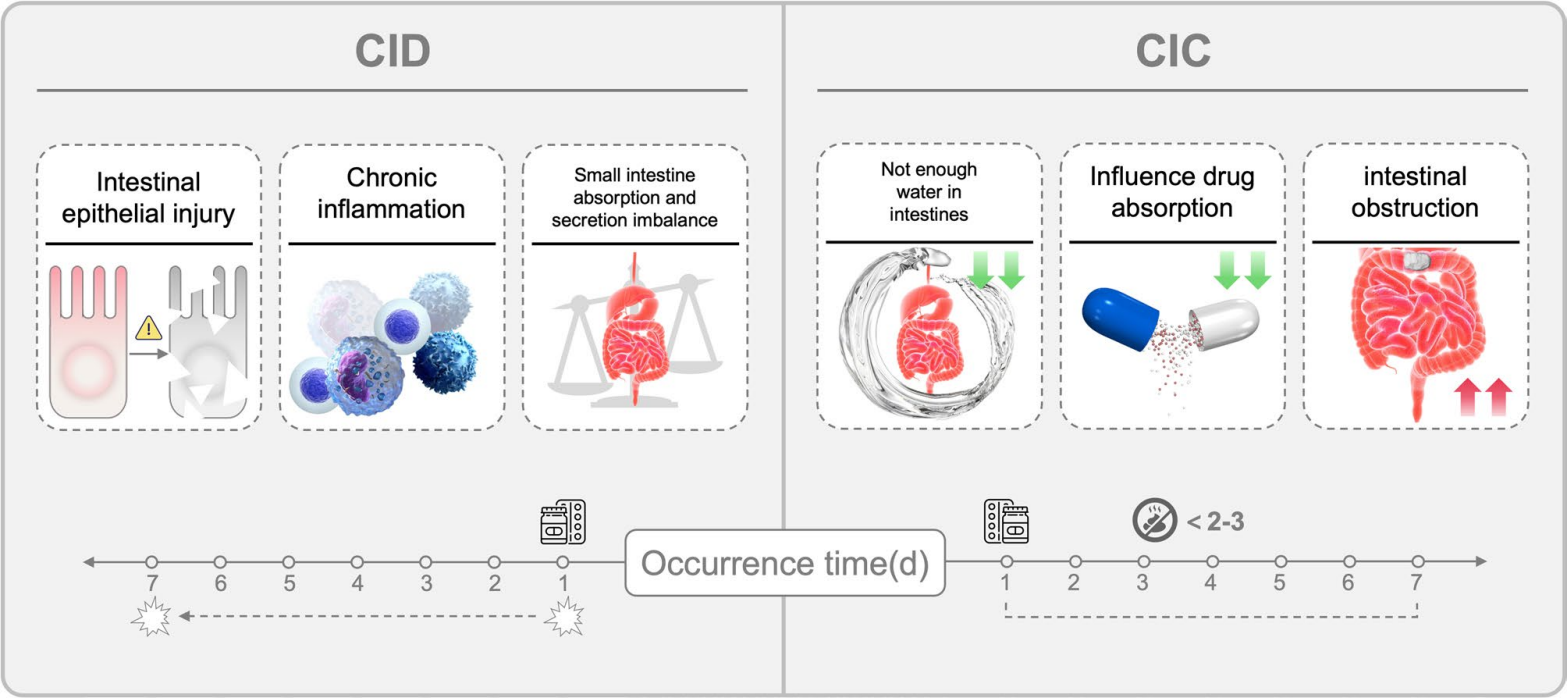
Pathophysiological Mechanisms and Onset Time of CID and CIC, Left (CID): CID is associated with intestinal epithelial injury, chronic inflammation, and small intestine absorption-secretion imbalance, leading to increased stool output. Right (CIC): CIC involves reduced water retention, impaired drug absorption, and intestinal obstruction, which delay bowel movements. Timeline: CID symptoms typically appear within 1–7 days post-chemotherapy, whereas CIC manifests within 2–3 days

Compared with children without constipation, children with acute lymphoblastic leukemia (ALL) experiencing CIC have increased mucositis, imaging, and overall healthcare utilization (Shafi and Bresalier 2010; Davila and Bresalier 2008). Constipation secondary to periwinkle alkaloids is a common concern in children diagnosed with leukaemia (Belsky et al. 2021) during chemotherapy. Patients often require additional medications, including chemotherapeutic drugs, antiemetic drugs, analgesic drugs, and sedative drugs, to treat their disease. To treat constipation produced by the above medications, laxatives and diaphoretics are again used to promote bowel movements. However, excessive use of diaphoretic medications can be counterproductive, leading to severe diarrhea. This excessive diarrhea leads to significant water loss from intestinal tract and overall exacerbates the difficulty of bowel movements again. Figure 2 provides a timeline representation of the onset of CID and CIC, demonstrating the differential progression of symptoms following chemotherapy administration.

#### Chemotherapy-exacerbated inflammatory bowel disease (IBD)

IBD patients undergoing chemotherapy are at an increase risk of severe CID and CIC, due to pre-existing mucosal inflammation and microbiota dysbiosis. Chemotherapy further disrupts tight junction proteins, such as claudin and occludin, and increases gut permeability, exacerbating diarrhea (Nakai and Miyake 2024). Additionally, inflammatory cytokines TNF-α and IL-6 are already elevated in IBD patients, making chemotherapy-induced colitis more severe (Voshagh et al. 2024).

## Chemotherapeutic agents causing CID or CIC

CID and CIC can result from the direct toxic effects of chemotherapeutic agents on the rapidly proliferating cells of gastrointestinal tract, as well as from secondary mechanisms such as neurotoxicity, altered mucosal integrity, and genetic predispositions affecting drug metabolism. This section focuses on the key chemotherapeutic agents known to cause CID and CIC, along with their mechanisms of action, side effects, and relevant genetic considerations. Table 2 presents a classification of key chemotherapeutic agents, their primary indications, mechanisms of action, and associated adverse gastrointestinal effects.

**Table 2:** Summary of mechanisms and side effects of different types of chemotherapeutic agents

Chemotherapeutic agent	Type	Indications	Main mechanism	Side effects
Irinotecan (CPT-11)	Topoisomerase I inhibitor	Colorectal cancer, lung cancer, lymphoma	Acetylcholinesterase inhibition leading to acetylcholine accumulation, causing severe diarrhea	High incidence (20–40%) of grade 3 or 4 diarrhea, delayed and dose-independent; SN-38 toxicity and apoptosis of intestinal cells
5-Fluorouracil (5-FU)	Antimetabolite	Digestive tumors (colon, stomach, oesophagus)	Inhibits thymidylate synthetase, preventing DNA synthesis	Diarrhea due to intestinal crypt cell apoptosis, neutrophil elastase inhibition, mucosal breakdown
Platinum-based Drugs	Alkylating agents	Ovarian, testicular, lung, colorectal cancers	Forms covalent bonds with DNA, inhibiting replication and transcription	Diarrhea associated with enteric neuropathy and motility disorders
Capecitabine	Antimetabolite	Metastatic colorectal and breast cancers	Converts to 5-FU in vivo, inhibiting DNA synthesis	Diarrhea, pancreatitis, colitis, ileitis, GVHD-like histological changes in intestines
Paclitaxel/Docetaxel/Cabazitaxel	Mitotic inhibitors	Breast cancer, lung cancer, ovarian cancer	Enhances polymerization of microtubule proteins, inhibiting mitosis and inducing apoptosis	Diarrhea (20–40%), with severe cases requiring hospitalization (especially with cabazitaxel), potential for Clostridium difficile infection
Vinca Alkaloids	Mitotic inhibitors	Lymphoma, leukemia	Neurotoxic effects impairing intestinal motility, leading to constipation and paralytic ileus	High rates of constipation (80–90%) with vinca alkaloids; common in pediatric oncology patients
Cyclophosphamide	Alkylating agents	Leukemia, solid tumors	Cytotoxic immunosuppressive effects altering mucosal integrity	Diarrhea or constipation due to intestinal mucosal ulceration

### Irinotecan (CPT-11)

Irinotecan (CPT-11) is a worldwide treatment for lymphoma, colorectal cancer, and lung cancer. The incidence of grade 3 or 4 CID in patients with irinotecan ranges from 20 to 40%, severe CIGT has limited clinical use (Tang et al. 2019). Irinotecan’s late-onset diarrhea is independent to dose, the median duration is 6 days, while dose-related diarrhea occurs within 24 h of administration, affecting up to 10% of patients (Stein et al. 2010). CPT-11 is a type of acetylcholinesterase inhibitor that accumulates acetylcholine in the neuromuscular and synaptic junctions, inhibiting acetylcholinesterase and causing severe diarrhea (Abraham and Sellin 2007). Alternations in adhesion molecule expression may directly contribute to the loss of mucosal layer integrity in mucositis (Al-Dasooqi et al. 2017). The ileum, cecum, and colon are the main areas of CPT-11 absorption, with the highest CPT-11 exposure values in the ileum (Shi et al. 2017; Kurita et al. 2011; Thiagarajah and Verkman 2012).

Genetic variations (polymorphisms) in the UGT1A1 gene can lead to differences in the activity of the UGT1A1 enzyme. One well-known genetic variant is UGT1A1*28, the occurance of neutropenia and CID resulting from elevated SN-38 in response to gene exposure following irinotecan dosing as affected by UGT1A1*6 and UGT1A1*28 allele conditions (Man et al. 2018; Nelson et al. 2021). UGT1A1*28 is associated with reduced enzyme activity, leading to slower metabolism of irinotecan and potentially higher levels of the active, toxic SN-38 in the body. Emma Hulshof conducted an experiment where patients administered irinotecan were genotyped at the outset, followed by an intentional 30% reduction in dosing for UGT1A1 poor metabolizers (UGT1A1 PMs). This adjustment was found to decrease the occurrence of febrile neutropenia in UGT1A1 PMs (Hulshof et al. 2022). Irinotecan is initially activated and converted to SN-38, however, SN-38 is a potent toxin, and the UGT1A1 enzyme plays an essential role in detoxifying it by adding a glucuronide group to SN-38, making it less harmful and easier to excrete from the body (Thiagarajah and Verkman 2012).UGT1A1 genotype-guided dosing not only provides a substantial improvement in individual safety but also offers some cost savings. This approach can also help alleviate CID.

De-conjugation of SN-38G to SN-38 is also associated with late-onset diarrhea. Irinotecan will combine with SN-38 to induce apoptosis and activation of inflammatory vesicles through the production of reactive oxygen species (ROS) and caspase-1-dependent pro-IL-1, pro-IL-18, and pro-IL-33 to become fully-fledged cytokines. Resident cells express inducible nitric oxide synthase (iNOS) and cyclooxygenase-2 (COX-2). iNOS and COX-2 are highly immunoexpressed in irinotecan-induced intestinal mucositis, and prostaglandins and nitric oxide produced by this process damage the gut (Ribeiro et al. 2016; Javle et al. 2007; Lima-Júnior et al. 2012). It has been speculated that CID with irinotecan correlate with the increase in beta-glucosidase (β-GLU)-producing bacteria (Stringer et al. 2008). To demonstrate that SN-38 rendered incapable of binding to SN-38G, Akinobu Kurita studied induced diarrhea with CPT-11 in a mouse model genetically lacking the UGT1A gene, known as the Gunn rat. In this model, β-GLU was unable to de-conjugate SN-38 in the rat. After administration of penicillin G and streptomycin (SM), SM alleviated diarrhea in Gunn rats, indicating that its effects are not mediated by β-GLU activity inhibition (Kurita et al. 2011).

### 5-Fluorouracil (5-FU)

5-FU is a chemotherapeutic agent that is commenly used in patients with digestive tumors, generally including colon, stomach, and esophagus cancer (Ribeiro et al. 2016). It is a pyrimidine antagonist antimetabolite and is phosphorylated to 5-FdUMP/5-FUMP. The main mechanism of 5-FU action is to inhibit thymidylate synthetase, thereby reducing the formation of thymidine monophosphate and preventing DNA synthesis.

Studies have confirmed that elevated levels of neutrophil and neutrophil elastase in the intestinal tract of mice can inhibit aquaporins 5/8 (AQP 5/8) expression, thereby reducing diarrhea symptoms and pathological damage (Sakai et al. 2014). Through intestinal capsule endoscopy, Kazuhiro Ota et al. found that intestinal mucosal damage from fluoropyrimidine was associated with the severity of CID, and intestinal mucosal rupture was more common in patients with CID with fluoropyrimidine (Ota et al. 2019). 5-FU chemotherapy could result in intestinal crypt cell apoptosis, specifically through inflammatory cytokines upregulation leading to NADPH oxidase 1-derived ROS production (Logan et al. 2009). Many chemotherapy regimens, including fluoropyrimidine, cause diarrhea and associated small bowel mucosal breakdown in patients. The degree of small bowel mucosal breakdown is more severe in patients receiving intravenous therapy than oral therapy (Ota et al. 2019).

Tertiary butylhydroquinone (TBHQ) significantly decreased lactate dehydrogenase (LDH) release, leading to cell death, while enhancing the proliferative capacity of human intestinal epithelial cells (HIECs) treated with 5-FU. Furthermore, TBHQ also significantly reduced the release of LDH and cell death in 5-FU-treated HIECs, and increased their proliferative capacity, as well as reduced diarrhea scores in the mice. It was concluded that TBHQ markedly impeded the activation of iron mutations, that 5-FU-induced intestinal mucositis was inhibited, and TBHQ could be used as a potentially novel intestinal mucositis protective agent (Deng et al. 2021).

The highest incidence of diarrhea occurs in patients who take irinotecan and bolus 5-FU weekly. The combination of CPT-11 and 5-fluorouracil would induce higher possibility of chemotherapy-induced diarrhea (Benson et al. 2004). Although the combination of oxaliplatin and 5-fluorouracil is a more effective chemotherapy regimen, it is also linked to a higher incidence of CIGT comparing with the use of 5-fluorouracil only. Conversely, the combination of capecitabine and oxaliplatin was less likely to result in severe diarrhea compared to the first two, but the risk remains higher than the use of the drug alone (Lee et al. 2014). Some patients are more likely to develop diarrhea after taking certain medicines because of a deletion or mutation in some of the genes.

### Platinum-based drugs

80–90% CIC rates occur after the administration of cisplatin, thalidomide, and periwinkle alkaloids (Wu et al. 2021; Gao et al. 2021). Platinum-based drugs, including cisplatin, oxaliplatin, and carboplatin, are chemotherapy regimens commonly used in clinical practice for managing various malignancies, including ovarian, testicular, lung, and colorectal cancers. They exert cytotoxic effects through the formation of covalent bonds with DNA, leading to DNA cross-linking and inhibiting DNA replication and transcription. Although these drugs have significant anti-tumor activity, they also have some significant side effects, with CID being a major concern (Stojanovska et al. 2015). Research has shown that chronic cisplatin treatment is associated with intestinal endothelial neuron loss and amplitude increase of nerve-induced contractions in gastric strips from mice, leading to episodic diarrhea. Thus, enteric neuropathy may serve as the underlying mechanism for chemotherapy-induced gastrointestinal motility disorders (Pini et al. 2016).

### Capecitabine

Capecitabine is an antimetabolite fluoropyrimidine deoxyribonucleoside carbamate that converts to 5-FU in vivo, it is used to treat metastatic colorectal and breast cancers, with common adverse reactions including gastrointestinal (GI) discomfort, abdominal pain, fatigue, hair loss and leukopenia. Diarrhea caused by Capecitabine is a common GI reaction caused by chemotherapy drugs (Khan et al. 2022; Mikhail et al. 2010). Recent studies have shown that capecitabine can induce pancreatitis (Yucel and Warmerdam 2010), small bowel colitis (Shao et al. 2022), and terminal ileitis (Zou et al. 2021; Hellemond et al. 2018) in locally advanced colorectal cancer patients. It was found that capecitabine also caused graft-versus-host disease-like histological changes in intestinal tissue biopsies of patients with colorectal cancer that improved after dose reduction or drug discontinuation (Ojukwu et al. 2023).

### Paclitaxel (paclitaxel/albumin paclitaxel/docetaxel/cabazitaxel)

Diarrhea caused by paclitaxel mainly occurs in patients with breast and lung cancer, and sometimes leads to alternating diarrhea and constipation. Previous studies have shown that paclitaxel in combination with carboplatin can result in Clostridium difficile infection diarrhea (Kamthan et al. 1992; Yamazawa et al. 2001). Numerous clinical case reports have also demonstrated the potentially high toxicity of paclitaxel to the intestinal tract. B.R. Loman et al. discovered that paclitaxel alters the gut microbiota, affects colonic tissue integrity, and causes microglia activation and fatigue through the brain-gut-microbiota axis in female mice (Loman et al. 2019). Makoto Furonaka et al. reported neutropenic fever and haemorrhagic diarrhea in a patient following treatment with paclitaxel and carboplatin combination in their clinical work (Furonaka et al. 2005). Additionally, Jayakody S et al. described the development of rectal perforation in advanced ovarian cancer patient after receiving the administration of paclitaxel followed by rectal perforation (Jayakody et al. 2018). Furthermore, Z Gastroenterol reported that a 58-year-old patient with metastatic squamous lung cancer developed fever, diffuse abdominal pain, and diarrhea when treated with paclitaxel in combination with radiotherapy (Rexroth et al. 1998).

Docetaxel is widely utilized in the treatment of malignant tumors, including breast cancer, non-small cell lung cancer, gastric cancer, and ovarian cancer. It is a semi-synthetic paclitaxel antitumor agent, which can enhance microtubule protein polymerization, inhibit cancer cell mitosis, and thus induce apoptosis. CID is a common adverse effect associated with docetaxel, occurring in 20%-40% of patients, with severe cases reported in 5–6% (Lee et al. 2013). Recent researches have shown that docetaxel-treated CID correlates with the duration of systemic blood levels, rather than with intestinal accumulation or the route of administration (Hendrikx et al. 2020).

Cabazitaxel-treated CID rate for metastatic desmoplasia-resistant prostate cancer was reported to be 47%. Grade 3–4 CID was observed in 6% of patients and required hospital admission for treatment. (Sperlich and Saad 2013).

### Vinca alkaloids (vinblastine /vinorelbine /vincristine)

Chemotherapeutic drugs such as vincristine analogues, which operate through a neurotoxic mechanism, cause a decrease in gastrointestinal smooth muscle stress and diminished gastrointestinal motility. Vincristine analogues are used in the treatment of lymphoma and leukaemia, and they can affect and impair the motility function of the intestines, leading to constipation and paralytic intestinal obstruction. These effects are more likely to occur in the elderly or with high doses of vincristine. Symptoms appear within 3 days and resolve within 2 weeks of administration, or present with peripheral neuropathy. Constipation due to vincristine is a frequent side effect in paediatric cancer patients (Belsky et al. 2020) and has been reported to a lesser extent in adult cases of acute lymphoblastic leukaemia (Masoumi et al. 2019).

Oncology patients use chemotherapeutic drugs, antiemetics, analgesics, and sedatives to manage their disease conditions during chemotherapy (Deepak and Ehrenpreis 2011), and constipation secondary to pergolide alkaloids is a common problem in children with leukemia (Belsky et al. 2021). However, 80–90% CIC rates have been reported after the administration of cisplatin, thalidomide, and vinca alkaloids (Vinblastine, Vincristine, and Vinorelbine) (Ghobrial and Rajkumar 2003).

### Cyclophosphamide

**Fig. 3:**
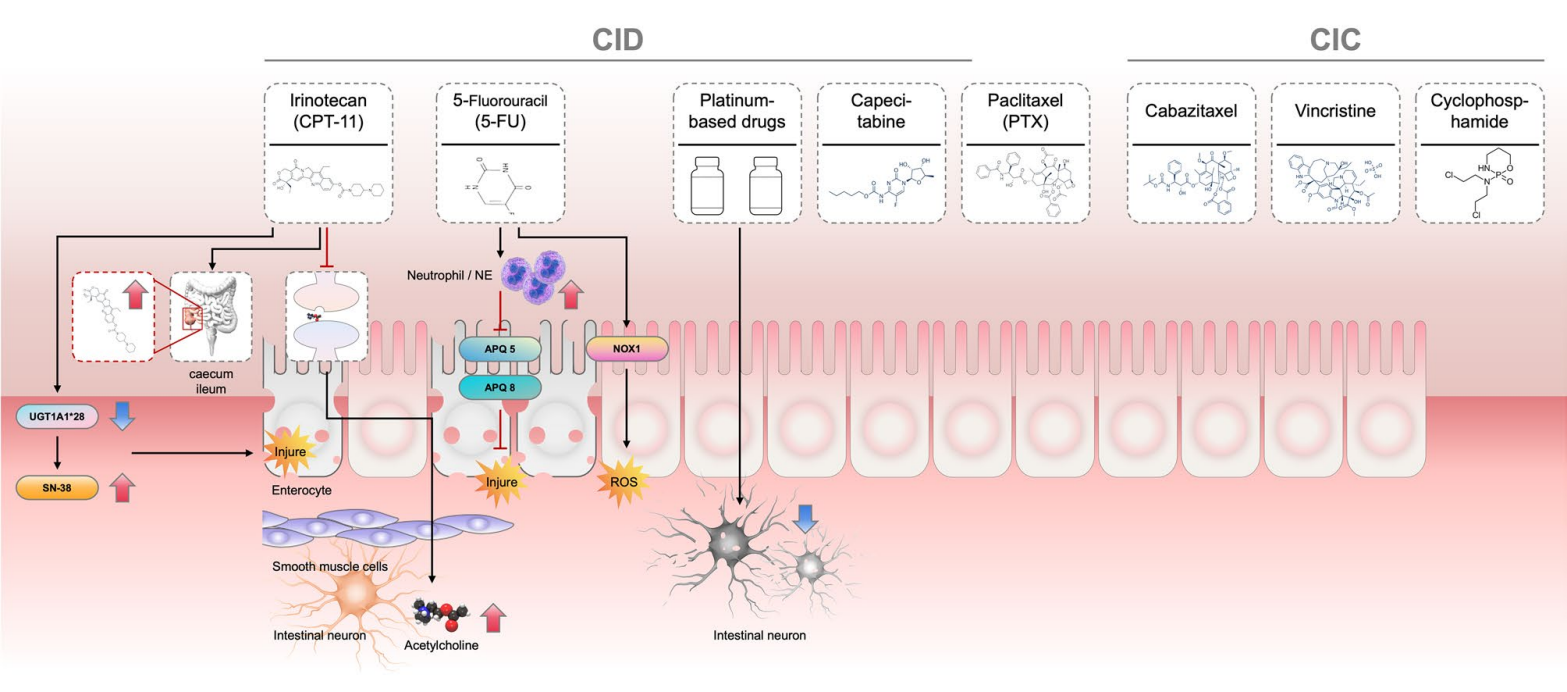
Schematic representation of the mechanisms of chemotherapy-associated diarrhea and constipation. Left (CID): Irinotecan and 5-FU contribute to CID via SN-38 accumulation, UGT1A1*28 polymorphism effects, intestinal epithelial damage, and neutrophil elastase activation. Platinum-based drugs also contribute to reactive oxygen species production, further damaging epithelial integrity. Right (CIC): Vinca alkaloids (e.g., vincristine) and taxanes (e.g., cabazitaxel) impair intestinal neuronal function, leading to reduced motility and constipation. These drugs inhibit acetylcholine release and smooth muscle contraction, ultimately causing intestinal obstruction

Cyclophosphamide is an alkylating anti-tumor agent, a cytotoxic immunosuppressive drug with broad-spectrum anti-tumor effects, effective against leukaemia and solid tumors. It affects the intestinal mucosa, leading to ulceration, which in turn alters water absorption and secretion, leading to diarrhea or constipation. The impact of chemotherapy on intestinal epithelial cells, enteric neurons, and inflammatory pathways is depicted in Fig. 3.

## Diagnosis of CIGT

The grading of CID in the ECOG version of the Common Toxicity Criteria (CTC) serves as a reference for determining the severity of diarrhea associated with all anticancer treatments (Andreyev et al. 2014). The grading system of CID in the CTC can be used as a reference for the severity of diarrhea associated with all anti-cancer treatments (Bossi et al. 2018).

The official grading of diarrhea status was released in November 2017. In the Common Terminology Criteria for Adverse Events (CTCAE) version 5.0, primary symptoms consist of elevations in the frequency of daily stools times above baseline along with increases in ostomy output comparing with baseline. Secondary symptoms include an increase in bowel movement frequency to four to six times daily from baseline, along with a moderate increase in ostomy output in comparison to usual level. Severe grade involves the onset of life-threatening symptoms that require hospitalization with urgent intervention (Health NIo. Common Terminology Criteria for Adverse Events (CTCAE) 2017).

The 2018 ESMO guidelines for adults with cancer diarrhea recommended to monitor the patient’s white blood cell and platelet profile, as a reduction in platelets can lead to coagulation dysfunction and thus gastrointestinal bleeding, with multidisciplinary team assessment of treatment and close monitoring if necessary (Andreyev et al. 2014).

CIC is an adverse reaction of the digestive system caused by chemotherapy, with an incidence of 16–48%. The occurance of constipation among patients with advanced cancer ranges from 40 to 90%, but its therapeutic need is often underestimated due to the variable diagnostic criteria for constipation, which primarily depend on the patient's complaints (Montemurro et al. 2016). The CIC does not currently have grading criteria for oncology patients, and the EMSO guidelines recommend that all cancer patients should undergo assessments for constipation. For advanced cancer, the factors contributing to constipation are typically longstanding and can be modified. Routine evaluation of the bowel function is essential, whether the patient is receiving treatment. Several pharmacological agents are used to manage CID and CIC. Loperamide, octreotide, and amitriptyline are commonly prescribed for CID, whereas polyethylene glycol, linaclotide, and elobixibat are indicated for constipation (Table 3).

**Table 3:** Pharmacological management of chemotherapy-induced diarrhea and chemotherapy-induced constipation

Drug	Mechanism of action	Usage	Potential side effects
Loperamide	Binds to opioid receptors in the gut to slow bowel movements	Acute and chronic diarrhea, CID	Constipation, abdominal discomfort, cardiac toxicity (Eggleston et al. 2020)
Octreotide	Inhibits release of intestinal hormones to reduce diarrhea	Severe, persistent CID	Nausea, abdominal discomfort, blood sugar changes (Lamberts and Hofland 2019)
Amitriptyline	Inhibits TLR2 and TLR4 activities to prevent colonic apoptosis	CPT-11-induced diarrhea	Sedation, dry mouth, constipation (Brueckle et al. 2023)
Polyethylene Glycol	Increases water in the stool for easier passage	Chronic idiopathic constipation	Nausea, cramping stomach, diarrhea (Dabaja et al. 2023)
Linaclotide	Increases intestinal fluid secretion and transit	Chronic constipation	Diarrhea, dehydration (Bassotti et al. 2018)
Elobixibat	Inhibits transportation of ileal bile acid	Functional constipation and CIC	Diarrhea, abdominal pain (Nakajima et al. 2022)

## Treatment options for CID or CIC

### Drug therapy

#### Clinical drugs commonly used for CID or CIC

Loperamide is a synthetic opioid analog utilized in the treatment of disease, primarily with its compound, loperamide hydrochloride. It is mainly used for conditions such as acute diarrhea and chronic diarrhea of various etiologies, and high doses of loperamide are commonly used for acute diarrhea caused by chemotherapy. Although loperamide may cause heart damage, it is still used clinically because the incidence is low, and the benefits of treatment generally outweigh the risks (Nakajima et al. 2022). Loperamide is an antidiarrheal drug that works primarily by binding to opioid receptors on the intestinal wall. This binding slows down intestinal muscle contractions, thereby reducing faecal movement and enhancing water absorption and electrolytes in intestines (Lemos et al. 2018). Loperamide is widely employed to relieve the symptoms of acute and chronic diarrhea, including CID, helping to decrease the frequency and urgency of bowel movements, making the stools more formed. However, its efficacy can differ from individual to individual, and the dosage of loperamide may need to be adjusted depending on the severity of the diarrhea and the individual's response (Andreyev et al. 2014). It is important that patients follow their doctor's recommended dosage and avoid overdosing, as an overdose can result in side effects including CIC, abdominal discomfort, and possible cardiac arrhythmia (Wu and Juurlink 2017). While receiving treatment with loperamide, patients may also receive dietary advice and hydration guidelines to help relieve symptoms and support overall health during cancer treatment. When dietary changes or dose adjustments of loperamide do not adequately control severe CID, other opioids may be considered, usually for patients with persistent, severe, or refractory diarrhea. More potent opioids such as morphine, codeine, or diphenhydramine/atropine slow bowel movements and reduce diarrhea. They all work essentially similarly to loperamide, by attaching to opioid receptors in the gut. Nevertheless, caution needs to be taken as opioids and can have side effects including constipation, sedation, nausea, and potential dependence or addiction (Wu and Juurlink 2017). Patients using opioids for CID should be observed for these side effects, instructed on how to manage them.

Octreotide is a synthetic analogue of somatostatin, a hormone that regulates various physiological processes, including hormone secretion and gastrointestinal motility. In the management of CID, octreotide primarily acts to inhibit the release of various hormones and neurotransmitters in the intestinal tract, thereby helping to reduce diarrhea (Wu and Juurlink 2022). Octreotide is usually used when CID is severe, persistent, or ineffective in response to other treatments such as antidiarrheal drugs or dietary modifications. Octreotide may be given in different ways, including subcutaneously or intravenously, based on the patient's condition and the advice of their healthcare provider. Subcutaneous octreotide is suggested for Grade 2 diarrhea and could also be contemplated for patients with Grade 1 diarrhea who have received loperamide without responding to the treatment (Mitchell 2006). However, octreotide may cause side effects, including nausea, abdominal discomfort, and changes in blood sugar levels (Maroun et al. 2007). It is important to monitor and manage these side effects during treatment. High doses of loperamide and octreotide are commonly advised for managing CID by irinotecan. However, loperamide is linked with notable rates of treatment failure in clinical settings, while octreotide efficacy needs further discussion. An increasing body of recent researches indicates that herbs and the extracted plant compounds could serve as beneficial complementary treatments for CID by irinotecan (Tang et al. 2019).

Amitriptyline is the most commonly used tricyclic antidepressant in the clinic, and animal experiments have preliminarily proven that small doses of Amitriptyline can prevent CPT-11-induced premature diarrhea with colonic apoptosis by inhibiting TLR2 and TLR4 activities (Bhattacharya S, Vijayasekar C, Worlding J, Mathew G 2009). Clinical drug therapies such as loperamide, octreotide, and amitriptyline are essential tools in managing CID and CIC, offering relief through various mechanisms. While these drugs are effective, they can cause potential side effects, and their use must be carefully monitored. Personalized dosing and combination with other treatment modalities may enhance outcomes and minimize risks.

The American Gastroenterological Association and the American College of Gastroenterology recommend the use of polyethylene glycol, sodium pyridinium sulfate, linaclotide, procalcitonin, and procalcitonin for the treatment of chronic idiopathic constipation, with a conditional recommendation for the use of fiber, lactulose, senna, magnesium oxide, and lubiprostone (Fakiha et al. 2019). Clinical pharmacists at Michigan Medicine Oncology have been providing symptom management to patients independently through the Collaborative Drug Therapy Management program, to develop protocols for the management of CIGT resulting from patient's cancer diagnosis or cancer therapy, and to enhance the quality of care (Chang et al. 2023). Daily administration of 72 µg linaclotide was found to significantly improve CIC symptoms, with a reduced rate of discontinuation due to diarrhea within treatment (Homan et al. 2021).

Elobixibat, a locally-acting ileal bile acid transporter protein inhibitor, accelerates colonic transit time and resolves constipation in short term. Both short- and long-term treatments are well tolerated, and clinical trial evidence supports this approach to enhance the concentration of endogenous bile acids in the colon for the treatment of chronic constipation (Schoenfeld et al. 2018). In this trial, patients with functional constipation or constipation-predominant irritable bowel syndrome were recruited. The SGLT1 inhibitor, mizagliflozin, demonstrated good efficacy and tolerability at doses of 5 and 10 mg functional constipation (Nakajima et al. 2018).

Prophylactic enteral defecation regimen can prevent constipation in patients with enteral feeding, while the usage may also increase the likelihood of diarrhea. A clinical analysis revealed that prophylactic laxative enteral protocols raised the risk of diarrhea, while not leading to a significant reduction in the risk of constipation compared to placebo (Correction to Lancet Gastroenterol Hepatol 2018). Additionally, a clinical trial was conducted to determine the effect of LactoCare synbiotic administration on patients experiencing CID, nausea, vomiting, and constipation in children with ALL receiving maintenance chemotherapy. In this study, children with ALL were randomized to receive either LactoCare synbiotic administration or placebo in two groups, the findings indicated that the incidence of constipation was significantly lower in LactoCare treatment group than in the placebo group, and the usage of synbiotic supplementation reduced diarrhea rate (Hay et al. 2019).

Recent evidence suggests that JAK inhibitors, particularly pan-JAK inhibitors like tofacitinib, might play a role in restoring inflammatory intestinal microenvironment in patients with IBD and immunotherapy-induced colonic injury (Eghbali et al. 2023). These small molecules control inflammation driven by cytokine and have been studied for their ability to lessen severe diarrhea and colitis in cancer patients receiving immunotherapy (Honap et al. 2024). Although their use in CID is not yet widespread, further investigation into their therapeutic application in iatrogenic colonic injury is warranted.

#### Traditional Chinese medicine therapeutic for CID and CIC

Traditional Chinese medicine offers alternative and complementary therapies for managing CID and CIC, with several compounds showing potential in reducing chemotherapy-induced gastrointestinal symptoms. These therapies work through anti-inflammatory mechanisms and modulation of gut flora, offering a holistic approach to treatment. A descriptive study collected through the Patient Identification Form and the Holistic Complementary and Alternative Medicine (CAM) Questionnaire found that cancer patients commonly utilize CAM to manage CIC, with phytotherapy being the most frequently CAM type used (Gravina et al. 2024).

Various types of saponins and saponin-containing herbal plants exhibit anti-inflammatory or laxative activity. To validate the saponin-rich cochineal extract, it was demonstrated that SPA exerts its laxative effects in a Loperamide-induced model of chronic constipation by stimulating anti-inflammatory responses and muscarinic cholinergic modulation (Toygar et al. 2020). The dried roots and rhizomes of Aster, a traditional Chinese medicine with more than 2000 years of history, and Aster tiliaceae ethanol extract may alleviate constipation primarily by antagonizing acetylcholine and muscarinic receptor binding to alleviate constipation, inhibiting Ca2 + influx, and reducing inflammation (Wu et al. 2021). Shouhuitongbian capsule, which is a traditional Chinese medicine targeting Prkaa1, improved the intestinal barrier in mice with constipation (Kim et al. 2019), it corrected the dysbiosis of the intestinal microbiota and activated bacterial metabolite-mediated synthesis of intestinal serotonin 5-HT, thereby promoting intestinal motility (Sun et al. 2023; Bai et al. 2022). Taurine has also been found to be effective in treating loperamide-treated CIC by increasing gastrointestinal peristalsis (Lin et al. 2022). Additionally, yellow tea extract, specifically Astragaloside IV (Lee et al. 2019), altered intestinal microbiota composition, increasing the diversity and abundance of the community, and had a protective effect on loperamide-treated CIC in mice (He et al. 2020).

Studies investigating rhubarb extract (RE) effects for diphenoxylate-induced constipation have analyzed changes in the colonic mucosa and intestinal flora. These studies clarified that RE enhances mucus secretion in the colon through the recruitment of mast cells and the enhancement of histamine and acetylcholine (Ach) in the mouse colon. It also leads to the up-regulation of the mRNA expression of Bip and CHOP, as well as the down-regulation of the expression of the mRNAs of Xbp1 and Xbp1s, thus inducing endoplasmic reticulum stress in the colonic epithelium linked to shifts in intestinal flora diversity and short-chain fatty acid (SCFA) levels (Gao et al. 2021).

Hericium erinaceus, also known as Lion’s Mane mushroom, has shown potential in reducing chemotherapy-induced intestinal damage, especially in mouse models exposed to 5-FU. According to studies, its bioactive compounds may have neuroprotective and gut microbiota-modulating properties that prevent inflammation and mucosal atrophy (Cao et al. 2021). This indicates its potential as a supplemental treatment for CID, though further clinical trials are needed to confirm its effectiveness in human.

In terms of water channel proteins, inhibitory neurotransmitters (nitric oxide, nitric oxide synthase, vasoactive intestinal peptide, and arginine pressin], excitatory neurotransmitters (substance P and gastrokinesis) levels, and morphological differences in colonic histology, studies suggest that supplementation with bacterial cellulose, and the consumption of mulberries may be effective in the alleviation of constipation (Gravina et al. 2023; Zhai et al. 2018). Some researchers have also elucidated the effects of Shenhuang poultice using a homozygous mouse model and found that it attenuated paclitaxel-treated CIC and intestinal morphologic injury while increasing paclitaxel effect on cytokine expression within the TLR4 pathway and IL-1β (Hu et al. 2019). Additionally, root bark tannins from E. cavea have been found to be effective in alleviating constipation and intestinal morphologic damage through modulation in membrane water channels expression, gastrointestinal hormones, the mAChR signaling pathway, and fecal microbiota to improve the constipation phenotype (Shi et al. 2020).

Additionally, quercetin enhances gastrointestinal motility and mucin secretion by regulating downstream signaling of mAChR (Kim et al. 2021). Treatment with the protease-activated receptor 2 agonist, SLIGRL-NH2, induced a significant increase in PAR-2 expression, and the number of mesenchymal Cajal cells also increased. High doses of SLIGRL-NH2 showed anti-constipation effects similar to those of prucalopride (Kim et al. 2018). Naringenin, a naturally occurring flavonoid commonly found in citrus fruits and tomatoes, alleviates loperamide-treatd CIC by increasing mesenchymal cellular levels of Cajal markers (c-Kit and SCF) and aquaporin 3 (Zhang et al. 2018).

In recent years, traditional Chinese medicine has also shown therapeutic potential for CID. Pueraria lobata Tang has been shown to reduce irinotecan-treated CID and decrease the abnormally increased levels of pro-inflammatory cytokines IL-1β, COX-2, ICAM-1 and TNF-α in mice (Yin et al. 2018). Semixia diarrhea heart soup and Scutellaria baicalensis soup have been found to reduce symptoms of gastrointestinal mucositis and delayed diarrhea in patients with colorectal cancer during chemotherapy courses (Wu et al. 2019; Chen et al. 2018). Sennoside injection improves CID and CIC in patients with non-small cell lung cancer (Lu et al. 2018). Shenzhu capsule significantly increased body weight and thymus/spleen index, reduced diarrhea, decreased colonic histopathological damage and restored the abnormal intestinal flora caused by CID in rats (Chen 2018). Table 4 summarizes non-pharmacological treatment approaches for chemotherapy-induced diarrhea (CID) and chemotherapy-induced constipation (CIC), highlighting their mechanisms and reported effectiveness.

**Table 4:** An overview of the non-pharmacological treatments

Treatment	Mechanism	Effectiveness
Probiotics	Modulate gut microbiota	Reduces CID
Dietary Modifications	Balance of fiber, vitamins, and low-fat intake	Reduces CID severity
Non-absorbed sugars	Modulates gut microbiota, enhances short chain fatty acid production (Wang et al. 2020)	Alleviates constipation
Transcutaneous Acupoint Electrical Stimulation (TAES)	Stimulates acupoints to relieve constipation	Effective in cancer-related constipation (Mao et al. 2021)
Auricular Acupressure (Jiang et al. 2023)	Stimulates ear points to relieve constipation	Effective in CIC

### Non-pharmacological treatments

Non-pharmacological treatments, including probiotics, dietary modifications, and biofeedback technology, provide additional strategies for managing CID and CIC. These treatments are generally well-tolerated and can be used in conjunction with pharmacological therapies to improve patient outcomes and life quality.

#### Probiotics for CID and CIC

Experiments have demonstrated that disrupting the microbiome with antibiotics prior to CIGT and affects mucosal recovery, while concurrently increasing the severity of diarrhea. This not only validates the link between the microbiome and gastrointestinal toxicity but also supports the conclusion that autologous faecal microbiota transplantation can accelerate mucosal recovery (Jiang et al. 2023). Several clinical studies have demonstrated probiotics' efficacy in preventing and managing gastrointestinal toxicity without significant adverse effects (Wardill et al. 2021; Thomsen and Vitetta 2018). Denise Csendes et al. identified that the abundance of intestinal flora predicted the neurological symptoms, body weight changes, and constipation in breast cancer patients treated with chemotherapy, through a meta-analysis of toxic side effects (Secombe et al. 2019). Given the safety and efficacy of probiotics, recent studies have demonstrated that they can reduce constipation and related complications caused by chemotherapy. A clinical study by the Department of Gastrointestinal Surgery at the Second Affiliated Hospital of Nanchang University in patients undergoing chemotherapy for colorectal cancer found that probiotics reduced the incidence of diarrhea and enhanced the presence of beneficial bacteria, including Bacteroidetes and Ascomycetes in intestinal microbiota after chemotherapy (Csendes et al. 2022). In their research, Sixteen patients with eligible colorectal cancer received radical surgery and required subsequent chemotherapy. Patients were assigned randomly to receive a probiotic combination from the postoperative period until the conclusion of the initial chemotherapy session which is the probiotic group, while the remaining half were given a placebo. During chemotherapy, gastrointestinal issues including nausea, acid reflux, abdominal pain, bloating, constipation, and diarrhea were documented. Analysis of stool samples collected before and after the postoperative cycle of chemotherapy showed that probiotic administration was effective in reducing chemotherapy-induced gastrointestinal complications (Csendes et al. 2022). A trial conducted after colorectal cancer surgery revealed that probiotics can improve the intestinal microenvironment and significantly reduce the levels of pro-inflammatory cytokines, including TNF-α, IL-6, IL-10, IL-12, IL-17A, IL-17C, and IL-22, compared to levels before treatment (Huang et al. 2023). Additionally, another meta-analysis of 1,024 oncology patients demonstrated that adding probiotics to conventional symptomatic treatment significantly reduced the overall rate of diarrhea and severe diarrhea, and shortened diarrhea duration in cancer patients (Zaharuddin et al. 2019).

Probiotics can improve gut microbiota and the incidence of CID can also be effectively prevented by taking probiotics before or during chemotherapy. Furthermore, probiotics can enhance the effectiveness of the combination of chemotherapy drugs (Zaharuddin et al. 2019; Lu et al. 2019). Lactobacillus and Bifidobacterium commonly found in probiotics, can induce anticancer effects by increasing apoptosis in cancer cells and reducing oxidative stress. Supplementation with Bifidobacterium probiotics has the potential to decrease chemotherapy-induced mucositis and diarrhea. Bifidobacterium can also reduce adverse effects after chemotherapy treatment by inhibiting pro-inflammatory cytokines (Bowen et al. 2007). Fecal transplants therapy also belongs to the broader category of intestinal microecological therapy. The alleviating effect of probiotics on constipation is related to gastrointestinal transport rate, fecal count and weight, serum and entero-gastrointestinal regulatory hormones, serum cytokines (Badgeley et al. 2021).

A research investigation assessing the impacts of probiotics on constipation induced by loperamide in Sprague–Dawley rats showed that probiotics helped alleviate a variety of symptoms induced by constipation, such as shortened intestinal transit time and suppression of intestinal flora disorders (He et al. 2022). However, the exact mechanism remains unknown. Interestingly, it has been reported that in contrast to earlier researches, the ability of Lactobacillus rhamnosus strains to improve constipation symptoms is not related to the levels of SCFAs in the colon (Wang et al. 2019). Lactobacillus lactis HFY14 (LLSL-HFY14) effectively improves constipation symptoms in Sprague–Dawley rats. Regulation of the VIP-CAMP-KA-AQP3 signaling pathway effectively inhibits isopropyl conjugated constipation like ltose, showing promise for bioavailability. LLSL-HFY14 subspecies was used to pre-treat mice with a broad-spectrum antibiotic to reduce the intestinal microbial content prior to paclitaxel treatment. Subsequently, intragastric tube feedings were administered in aseptic conditions to mice, which were sourced from the mice treated with either a vector or paclitaxel to obtain intestinal contents. The data suggest that changes in gut microbes associated with chemotherapy contribute to the onset of inflammatory responses and psychological effects (Inatomi and Honma 2021; Tan et al. 2021).

IBD patients receiving chemotherapy may require different CID/CIC management strategies, as routine antidiarrheal or laxative use could worsen underlying colitis or strictures. Probiotics Lactobacillus rhamnosus and Bifidobacterium breve have shown promise in protecting gut integrity in these patients (Grant et al. 2021; Gao et al. 2024).

A mouse model of constipation was induced by the application of difenocoumide by tube feeding to mice and patients. The study identified the impacts of Bifidobacterium animalis Lactobacillus subsp. Lactis (MN-Gup) on alleviating constipation in BALB / c mice and humans. By measuring SCFA concentrations change and microbial composition of feces, it was found that MN-Gup relieved constipation by promoting the growth of acetate-producing bifidobacteria Ruminoccaceae_UCG-002, and Ruminoccaceae_UCG-005 (Guo et al. 2023).

Li, Rong et al. identified a native strain of R. gnavus highly present in fresh fecal samples from individuals suffering from refractory constipation. They found that oral administration of R. gnavus antiplasmodialis effectively ameliorate constipation symptoms in constipated mice induced by loperamide and transplanted feces from constipated patients. Additionally, it significantly improved stress-related behaviors in mice (Wang et al. 2021).

Administering d-tagatose as a dietary supplement proved to be effective in prevention and alleviation of constipation in Kunming mice, showing its potential as a promising prebiotic candidate with properties that relieve constipation (Li et al. 2023). In this study, both the Japonica sugar by-products, soluble dietary fiber (SDF) and insoluble dietary [IDF), improved feces-related markers, gastrointestinal tract passage, and histological morphology in mice induced with loperamide by facilitating the biosynthesis of SCFAs. Nonetheless, SDF demonstrated superior efficacy in combating constipation, supported by its enhanced water retention capacity. In addition, SDF and IDF elevate the concentrations of antioxidant enzymes (SOD and GSH-Px), reinstated the presence of intestinal neurotransmitters, and preserved the functionality of tight junction proteins like ZO-1, JAM-1, and Occludin (Liang et al. 2019). Notably, SDF and IDF also enhanced the up-regulatory effect on Muribaculacea ratio, Prevotellaceaen and Lachnospiraceae, which is essential for maintaining intestinal immune homeostasis (Liang et al. 2019).

Probiotics have demonstrated effectiveness in reducing chemotherapy-induced diarrhea and improving gut microbiota balance. They are a valuable adjunct to conventional therapies, offering a low-risk option for enhancing gastrointestinal health in cancer patients.

#### Dietary modifications in the alleviation of CID and CIC

A descriptive study using multiple scales found that one-third of patients undergoing ambulatory chemotherapy experience constipation, with the condition being of moderate severity and moderately affecting their quality of life (Pehlivan and Nural 2022). Compared with the dietary counseling intervention group, the control group of patients with breast cancer experienced more malaise, nausea and vomiting, pain, dyspnea, loss of appetite, constipation, and diarrhea (Cao et al. 2023). Some studies have indicated that lower vitamin D levels are linked to an increased risk of severe diarrhea. The World Cancer Research Fund recommends a cancer prevention diet that is to eat more whole grains, vegetables, fruits, and legumes; eat less fast food; consume fewer red and processed meats; and drink fewer sugary beverages (Najafi et al. 2019). Referring to this recommendation, for a vegetable-based diet with controlled fibre content, researchers developed the Mediterranean Modified Health Diet (MMHD) and found that the MMHD was able to control the incidence of post-CID (Artale et al. 2022).

Dietary factors, such as dehydration and insufficient intake of fiber-containing roughage, often lead to gastrointestinal peristalsis difficulties and constipation in tumour patients. These patients frequently intake high-protein and high-fat foods to supplement their nutrition while neglecting the consume of dietary fiber, vitamins, and other laxative food, resulting in an imbalance in the patient's dietary structure (McQuade et al. 2016). Dietary modifications, particularly those incorporating high-fiber, vitamin-rich foods, can significantly alleviate CID and CIC symptoms. A balanced diet tailored to the needs of cancer patients can reduce the severity of gastrointestinal side effects and improve overall treatment tolerance.

#### Non-absorbed sugars

Non-absorbed sugars like lactulose and β-glucan offer effective constipation relief by modulating gut microbiota and enhancing intestinal motility. These agents are particularly beneficial for patients who prefer non-pharmacological approaches or who have not responded well to other treatments.

Lactulose is a frequently prescribed laxative that finds widespread use in clinically for constipation (Artale et al. 2022). Lactulose modulates the intestinal microbiota and intestinal metabolites to ameliorate loperamide-treated CIC constipation in mice by reversing the dysfunction of the intestinal microbiota and facilitating the production of intestinal metabolites, including bile acids and SCFAs. Additionally, various glycans have also been shown to prevent loperamide-treated CIC in mice by modulating the microbiota spectrum, including β-glucan, p-chitosan oligosaccharide, and konjac glucomannan (Zhang et al. 2021a).

β-glucan, a naturally occurring polysaccharide widely found in yeasts, fungi, and plants, influences the intestinal flora, restores the micro-ecological balance, regulates neurotransmitter expression and tight junction proteins, and restores the intestinal epithelial mechanical barrier (Zhang et al. 2021b). The mechanism of chitosan oligosaccharide (COS) amelioration of loperamide-treated CIC in mice with COS significantly inhibits ceramide glucosyltransferase, sphingolipid 4-desaturase, alkaline ceramidase 1, sphingosine kinase 2, lysophosphatidylcholine acyltransferase 1 and aromatic-L-amino acid decarboxylase (Chen et al. 2019).

In addition to these two sugars, other sugars also have potent effects. The beneficial effects of spirulina polysaccharide treatment include improved defecation, increased acethylcholinesterase (AchE) activity, decreased nitric oxide concentration, renewal of damaged intestinal villi, and effects on the expression of relevant genes in constipated mice (Zhang et al. 2022). The role of inulin polysaccharides in improving functional constipation has been investigated in conjunction with proteomic studies and intestinal flora analysis. Proteomics research showed that the polysaccharide of Clostridium morifolium reduces intestinal lesions, improves intestinal peristalsis, boosts the intake of amino acid, maintains intestinal homeostasis, and reduces constipation by controlling RAS expression, FA-binding protein 1, and solute carrier family 1 member 5 proteins (Ma et al. 2019).

#### Biofeedback technology

Biofeedback technology represents an innovative and non-invasive approach to managing cancer-related constipation. These methods are effective, safe, and can be incorporated into broader treatment plans to enhance patient comfort and outcomes. The Bristol Stool Form Scale and Constipation Assessment Scale scores of the Transcutaneous Acupoint Electrical Stimulation (TAES) were noticeably greater in the test group than in the control group that received only routine care. This suggests that TAES is a safe and useful nursing intervention that can successfully treat constipation in patients with non-small cell lung cancer after chemotherapy (Wang et al. 2020).

#### General treatment

General treatments provide an additional option for cancer patients to treat CIC. These non-invasive methods are accessible, safe, and can be integrated into comprehensive care plans to improve patient well-being. A randomized controlled trial demonstrated that auricular acupressure was helpful in alleviating constipation in breast cancer patients receiving chemotherapy. This involves the applying of pressure to particular areas on the ear corresponding to various body systems. It was also found that this non-invasive technique alleviated constipation and improved the overall comfort of patients. It was concluded to be a safe and acceptable nursing intervention (Mao et al. 2021; Wang et al. 2021).

## Advances in CIGT research

According to the available evidence, osmotic laxatives including polyethylene glycol can form a hypertonic state in the intestines to absorb water after oral administration, while simultaneously preventing the intestines from absorbing water, resulting in an increase in intestinal content volume, enhancing intestinal peristalsis, and causing defecation. Pro-dynamic drugs target intestinal nerve endings, trigger the release of motor neurotransmitters, antagonize inhibitory neurotransmitters, or stimulate directly on smooth muscle to increase intestinal motility. These drugs demonstrate notable efficacy in treating chronic transmission constipation, such as Plukapili. Guanylate cyclase-C agonists (representative drugs: Linaclotide) can alleviate the signs of chronic constipation including abdominal pain and constipation.

A trial in chronic constipation patients showed that a vibrating capsule was more effective than placebo in alleviating gastrointestinal symptoms and quality of life. The vibrating capsule was shown to be safe and well tolerated (Shin and Park 2018). Linaclotide activates the intracellular conversion of guanosine 5-triphosphate to cyclic guanosine monophosphate, thereby stimulating the secretion of intestinal fluid. Chloride channel activators, such as Rubiprost, can promote intestinal epithelial secretion, and upregulate natural defecation frequency. Although microecological agents are not the first-line drugs for the treatment of chronic constipation, they can correct the imbalance of intestinal flora, facilitate intestinal peristalsis, and aid in gastrointestinal motility recovery. Increasingly, researchers advocate for their use as a long-term adjunctive medicine for chronic constipation (Rao et al. 2023).

Andro significantly reduced the expression of caspase8/3 and 5-Fu-induced protein expression of Bax and the phosphorylation of p38. Additionally, Andro was able to adjust the restoration of cell viability that was diminished by 5-Fu. The inhibition of p38 MAPK mediates Andro’s anti-apoptotic effect on 5-Fu-induced intestinal mucosal inflammation, suggesting that Andro may have potential palliative complications in patients receiving 5-Fu chemotherapy (Rao and Brenner 2022). A novel 5-amino acid mixture has been suggested as potentially efficacious against antidiarrhea. Aman Chauhan and colleagues discovered that the amino acid mixture (enterade) may alleviate CIGT, as indicated by a retrospective chart review (Xiang et al. 2020).

More investigation has been done on the use of probiotics, prebiotics, or microbial transplantation treatments to prevent and cure behavioral disorders brought on by chemotherapy. The NeoFamily History Score (NeoFHS), which is calculated based on the kind of cancer, age at diagnosis, relative relationship, and number of affected relatives, acts as a useful and effective biomarker. Clinical prediction models may make it possible to effectively control constipation in chemotherapy patients. Additionally, in patients receiving neoadjuvant platinum-based chemotherapy for breast cancer, this biomarker predicts adverse outcomes related to chemotherapy, with a greater prevalence of constipation and diarrhea noted in individuals with higher NeoFHS (Chauhan et al. 2020). Adopting this therapeutic approach has the potential to completely transform the way cancer is treated, greatly enhancing patient satisfaction and lowering death rates.

## Conclusion

Pathophysiological factors of cancer, such as tumours located in the intestines or extrinsic bowel compression, contribute to complications like diarrhea and constipation. Individual differences in defecation habits, along with changes in the surrounding environment after hospitalization, can lead to physiological constipation (Xu et al. 2021). Patient factors such as anxiety, depression, prolonged bed rest, and insufficient activity, also play a significant role. The parasympathetic nervous system of patients is often inhibited due to the side effects of chemotherapy, excessive economic pressure, and a poor psychological state. Activity factors: Patients with tumours frequently experience generalized weakness due to the effects of disease and chemotherapy, and the associated reduction in activity and food intake, coupled with a lack of mechanical stimulation of the intestinal tract, leads to CIGT (Dinning and Lorenzo 2011).

In addition to the intestinal toxicities associated with chemotherapy, epidermal growth factor receptor-tyrosine kinase inhibitors have also been linked with drug-related toxicities such as diarrhea, acne-like rash, mucositis and onychomycosis, as evidenced in several phase 3 clinical studies (Gallegos-Orozco et al. 2012; Hsu et al. 2018; Migden et al. 2018; Gounder et al. 2018). CID was also the most common adverse event in a phase III clinical study of ibrutinib (Imbruvica) in combination with rituximab (Rituxan) for Waldenström's macroglobulinemia (Liu et al. 2018). A retrospective study of immune checkpoint inhibitors revealed that immunosuppression of diarrhea or colitis did not have a significant affect overall survival, and diarrhea emerged as an independent predictor of overall survival (Dimopoulos et al. 2018). These findings underscore the need for enhanced clinical monitoring and early warning of CID when using checkpoint inhibitors in combination with chemotherapeutic agents.

Opoid-treated CIC is prevalent among patients receiving opioids for cancer pain, and there is a large body of literature examining this condition. Gastrointestinal symptoms in cancer patients who are prescibed opioids alongside radiotherapy often lead to dose reductions, delays, and discontinuation of therapy. Symptoms such as chemotherapy-induced nausea, bloating, vomiting, constipation, and diarrhea can persist for up to 10 years after treatment (Wang et al. 2018). Thus, greater attention is required for managing CIGT.

CID and CIC represent significant and persistent challenges in the management of cancer patients undergoing treatment. Despite advances in supportive care, there remains no universally effective approach to balancing the treatment of these conditions. The current pharmacological interventions, while offering relief, often create a delicate balancing act, drugs designed to combat constipation may inadvertently exacerbate diarrhea, and those targeting diarrhea can sometimes lead to severe constipation. This highlights a critical gap in achieving an effective therapeutic strategy. While progress has been made, substantial gaps remain in fully deciphering the molecular pathways involved in CIGT. Specifically, the roles of the gut microbiome and inflammatory responses require further exploration to understand their contributions to both the development and mitigation of these toxic effects. Looking forward, the key to overcoming this challenge lies in the development of more personalized treatment approaches. Future research must aim to identify and integrate individual patient factors—such as microbiome diversity, metabolic rate, genetic predispositions, and overall physiological responses to chemotherapy. By tailoring treatments based on these unique patient characteristics, clinicians can better manage the complex side effects of chemotherapy, minimizing the risk of exacerbating one condition while treating another.

The future of CID and CIC management will likely involve a multidisciplinary approach, combining insights from oncology, pharmacology, genetics, and patient-centered care. Future research could consider how CID and CIC management strategies can be optimized for patients with pre-existing gut disorders, such as IBD, who may require tailored therapeutic approaches. This collaborative effort is essential to finding the balance point where treatments are both effective and tailored to individual needs, ultimately improving patient outcomes and quality of life during chemotherapy.

## Data Availability

No datasets were generated or analysed during the current study.

## Abbreviations

CIGT: Chemotherapy-induced gastrointestinal toxicity
CID: Chemotherapy-induced diarrhea
CIC: Chemotherapy-induced constipation
IBD: Inflammatory bowel disease
CTCAE: Common terminology criteria for adverse events
ALL: Acute lymphoblastic leukemia
CPT-11: Irinotecan
5-FU: 5-Fluorouracil
ROS: Reactive oxygen species
iNOS: Inducible nitric oxide synthase
COX-2: Cyclooxygenase-2
β-GLU: Beta-glucosidase
SM: Streptomycin
AQP: Aquaporins
TBHQ: Tertiary butylhydroquinone
LDH: Lactate dehydrogenase
HIECs: Human intestinal epithelial cells
GI: Gastrointestinal
COS: Chitosan oligosaccharide
CTC: Common toxicity criteria
CAM: Complementary and alternative medicine
RE: Rhubarb extract
Ach: Acetylcholine
TAES: Transcutaneous acupoint electrical stimulation
SCFAs: Short-chain fatty acids
LLSL: Lactobacillus lactis
SDF: Soluble dietary fiber
IDF: Insoluble dietary
MMHD: Mediterranean modified health diet
COS: Chitosan oligosaccharide
AchE: Acethylcholinesterase
TAES: Transcutaneous acupoint electrical stimulation
NeoFHS: NeoFamily history score
